# User-Driven Living Lab for Assistive Technology to Support People With Dementia Living at Home: Protocol for Developing Co-Creation–Based Innovations

**DOI:** 10.2196/10952

**Published:** 2019-01-28

**Authors:** Robin CP van den Kieboom, Inge MB Bongers, Ruth E Mark, Liselore JAE Snaphaan

**Affiliations:** 1 Tranzo Tilburg School of Social and Behavioral Sciences Tilburg University Tilburg Netherlands; 2 Research Unit Evidence Based Management of Innovation Mental Health Care Institute Eindhoven Eindhoven Netherlands; 3 Centre for Healthcare Management Erasmus University Rotterdam Netherlands; 4 Department of Cognitive Neuropsychology Tilburg School of Social and Behavioral Sciences Tilburg University Tilburg Netherlands

**Keywords:** dementia, family caregivers, longitudinal studies, technology

## Abstract

**Background:**

Owing to no cure for dementia currently, there is an urgent need to look for alternative ways to support these people and their informal caregivers. Carefully designed interventions can answer the unmet needs of both people with dementia and their informal caregivers in the community. However, existing products, systems, and services are often too complex or unsuitable.

**Objective:**

This study aims to identify, longitudinally, the changing needs (as dementia progresses) of people with dementia living at home and their informal caregivers. By developing co-creation-based innovations, these changing needs will hopefully be met.

**Methods:**

A user-driven Living Lab design is used to structurally explore the needs over time of people with dementia (and their informal caregivers) living in the community in the North Brabant region of the Netherlands. In addition, co-creation-based innovations will be developed, tested, and evaluated by these people and their caregivers at home. All participants will complete complaints-oriented questionnaires at 3 time-points—at the baseline, 1 year, and 2 years after they start participating. Home interviews are scheduled to explore if and how these complaints translate into participants’ specific needs or wishes. Focus groups meet on a monthly basis to further identify the needs of people with dementia and their informal caregivers and provide feedback to the stakeholders. In the context field, participants have an opportunity to actually test the products at home and provide feedback. Quantitative outcome measurements include neuropsychiatric symptoms, cognitive decline, independence in activities of daily living, safety, and caregiver burden. Qualitative outcome measurements include feedback to the stakeholders regarding the needs of people with dementia and their informal caregivers and how these needs change over time, as well as user experiences about the specific innovations.

**Results:**

Participant recruitment will start in September 2018 and is ongoing. The first results of data analyses are expected in the spring of 2019.

**Conclusions:**

The overall aim of Innovate Dementia 2.0 is to facilitate person-centered innovations developed for people with dementia and their informal caregivers at all stages as dementia progresses. This should lead to newly designed concepts and innovations, which are better able to answer the needs of people with dementia and their caregivers in the community.

**International Registered Report Identifier (IRRID):**

DERR1-10.2196/10952

## Introduction

According to the World Alzheimer Report, there were approximately 47 million people with dementia worldwide in 2016, and this number is expected to increase to 131 million by 2050 [1]. Dementia affects all aspects of a person’s life, and the progression is highly variable in individuals. As dementia progresses, people with dementia experience more problems in activities of daily living (ADL) such as dressing, toileting, and bathing [2]. Consequently, more responsibility is required from informal caregivers (eg, spouses, children, or friends), which can lead to increased stress and burden for them. Research indicates that informal caregivers of people with dementia experience higher rates of burden compared with other (nondementia) informal caregivers [3]. Owing to no cure for dementia currently, there is an urgent need to look for alternative ways to help and support people with dementia and their informal caregivers in the community [4].

Carefully designed interventions can answer the unmet needs of both people with dementia and informal caregivers at various stages as dementia progression. However, existing products, systems, and services are often too complex to be used by people with dementia [5]. The usability and adaptability of newly designed innovations, therefore, deserves more attention. To address this challenge, the Innovate Dementia project was set up in 2012 and was funded by Interreg IVB NEW [6]. The ambition of this project was to create a network of experts in the Northwest region of Europe who would enable the development of user friendly or user-based innovative solutions for people with dementia and informal caregivers living at home. Based on the Interreg program outline, 3 main challenges were defined as follows: to analyze the needs of people with dementia and their informal caregivers (in care and support); to develop innovative solutions; and to establish a sustainable collaboration between different stakeholders involved in dementia care innovations. By the end of this successful project in December 2015, several innovations had been developed and tested at home [7-9], and some have already been implemented into the daily care of people with dementia. After 2015, the European collaboration ended, and the Dutch partners continued the project under the name Innovate Dementia 2.0 (ID 2.0) as a sustainable regional collaboration. The current collaboration is between Geestelijke Gezondheidszorg Eindhoven en de Kempen (GGzE; Mental Health Care Institute Eindhoven), other health care organizations (Zuidzorg, Archipel), higher educational institutes (Tilburg University, Eindhoven University of Technology, and Fontys), business network Brainport, and governmental bodies. By using a Living Lab (LL) design [10] (see Methods for details), we primarily aim to explore, on a longitudinal basis, the changing needs of people with dementia and their informal caregivers in the community as dementia progresses. The secondary aim is to develop co-created (by innovators, people with dementia, and informal caregivers) innovations, which are tested and evaluated in real-life situations. To enhance the usability and adaptability of newly designed concepts, the involvement of people with dementia and their informal caregivers is crucial [10]. A user-driven LL design is an innovative method that allows for active user involvement in a realistic context [11]. A user-driven LL focuses on solving users’ specific problems in everyday life in a way that is consistent with the values and requirements of users and are sustainable because they are built around users [12]. Bharucha et al [13] underline the need to evaluate concepts in a real-life context when developing assistive technology for people with dementia. The ecological validity of the study also improves [14], as well as the likelihood that these innovative proposals will be adopted and implemented by business stakeholders [15], which is essential if these products are to be brought to the market. By using this design, people with dementia and informal caregivers are directly involved in each phase (from idea to concept to product to testing the innovations) to enhance the adaptability and usability of new products, services, and tools. This study describes the design and protocol of ID 2.0.

## Methods

### Study Design

**Figure 1:**
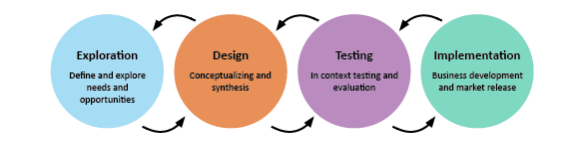
Stages in the development in a Living Lab design.

In the ID 2.0 LL, people with dementia and their informal caregivers have a central position in the development of innovations [16,17]. They are involved in the different stages of the development process; these stages include exploration, design, testing and evaluation, and finally implementation (Figure 1).

During the exploration stage, validated questionnaires, interviews during home visits, and focus group meetings are used structurally to explore the needs of people with dementia and their informal caregivers. At the design stage, these needs (gathered during the exploration stage) are used to find a suitable design concept during the focus group meetings. In the evaluation stage, a design proposal is evaluated in context-field studies on the potential of the innovation in the real-life setting and on its usability for people with dementia and their informal caregiver. In these field studies, participants use the innovations for a certain period of time (often 2-3 weeks) in the real-life setting to experience the product’s usability, feasibility, safeness, and provide feedback. In the implementation phase, further activities, including long-term testing, fundraising, and building production lines, are needed to bring a design to the market.

### Study Population

The study population comprises people with dementia and their informal caregivers, all living in the North-Brabant region of the Netherlands, who are recruited from the following institutions: Geestelijke Gezondheidzorg Eindhoven en de Kempen, Archipel and Zuidzorg (elderly federations); Alzheimer Nederland; centers for daytime activities for people with dementia; and through social media.

### Inclusion and Exclusion Criteria

The inclusion criteria are met if a person has the diagnosis of dementia (every subtype) and is living at home; there is a committed informal caregiver (spouse, family member, or friend) for people with dementia and both have sufficient understanding and mastery of the Dutch language. Of note, no exclusion criteria have been formulated.

### Measurements

#### Data Collection

In this ongoing initiative, quantitative data will be obtained using questionnaires to assess the cognitive, emotional, and behavioral complaints of both people with dementia and their informal caregivers.

The quantitative data will be used as a starting point to obtain further qualitative data to extract, in detail, the specific individual needs in the daily functioning of people with dementia and their informal caregivers. Furthermore, qualitative data will be obtained using home interviews, focus group meetings, and context-field evaluation (see below for details of all measurements procedures).

#### Background Information and Questionnaires

The following details will be obtained from a list of written questions regarding people with dementia: age, gender, marital status, the highest educational level achieved [18], previous employment, living situation, diagnosis, global staging of the severity, time since diagnosis, and comorbidity (Tables 1 and 2). In addition, informal caregivers’ age and their relationship (spouse, child, brother or sister, other family, friend, other specified) with persons with dementia will be obtained. An expert panel of 4 professional caregivers selected validated questionnaires to assess the social, emotional, cognitive, and physical status of people with dementia and the burden experienced by informal caregivers*.* Informal caregivers together with people with dementia fill in these questionnaires at 3 time-points—at the start of participation (T0), 1-year (T1), and 2-year (T2) follow-up.

**Table 1:** Demographics of people with dementia.

Sociodemographic variable and questionnaire		At the baseline	1-year follow-up	2-year follow-up
**Age, gender, marital status, education level, and former employment of people with dementia**				
	What is the date of birth of the person with dementia?	✓	✓	✓
	What is the marital status of the person with dementia?	✓	N/A^a^	N/A
	Highest level of education [18]	✓	N/A	N/A
	Former occupation of the person with dementia?	✓	N/A	N/A
**Living situation**				
	Current living situation?	✓	N/A	N/A
**Diagnosis**				
	Alzheimer’s disease/vascular dementia/Parkinson’s disease/frontotemporal dementia/other	✓	N/A	N/A
**Global staging of severity**				
	Clinical Dementia Rating Scale [19]	✓	✓	✓
**Time since diagnosis**				
	Date of diagnosis?	✓	N/A	N/A

^a^N/A: not applicable.

**Table 2:** Demographics of caregivers.

Sociodemographic variable	Variable or questionnaire	At the baseline	1-year follow-up	2-year follow-up
Relationship of caregiver with person with dementia	What is your relationship with the person with dementia?	✓	N/A^a^	N/A
Age of caregiver	What is the caregiver’s date of birth?	✓	✓	✓

^a^N/A: not applicable.

#### Social Emotion Status

To evaluate the neuropsychiatric symptoms of people with dementia, the Dutch version of the Neuropsychiatric Inventory Questionnaire (NPI-Q) is used [20]. The NPI-Q is a retrospective caregiver-informant questionnaire covering the following 12 domains: delusions, hallucinations, agitation or aggression, dysphoria or depression, anxiety, euphoria or elation, apathy or indifference, disinhibition, irritability or lability, aberrant motor behaviors, nighttime behavioral disturbances, and appetite or eating disturbances. The informal caregiver is asked to circle a “yes” or “no” in response to each question and to either proceed to the next question if the answer is “no” or to rate the symptoms severity in the last 4 weeks if the answer is “yes.” Severity is rated for each question as 1 (mild), 2 (moderate), or 3 (severe). The total NPI-Q severity score represents the sum of individual symptom scores and ranges from 0 to 36. Caregiver distress associated with the symptom is rated on a 0- to 5-point scale. The total NPI-Q distress score represents the sum of individual symptom scores and ranges from 0 to 60.

#### Background Information

##### Cognitive Status

To evaluate the cognitive decline of a person with dementia—from the viewpoint of a caregiver—the Dutch version of the Informant Questionnaire on Cognitive Decline in the Elderly is used at the baseline [21]. The Informant Questionnaire on Cognitive Decline in the Elderly is a questionnaire that asks informal caregivers about changes in an elderly person’s everyday cognitive function over the past 10 years. The questionnaire aims to assess the cognitive decline over these past 10 years. The cutoff scores are based on the total score divided by the number of questions (range 1-5). A score <3.00 indicates progression in cognitive functioning; a score of 3.00 indicates no change; 3.01-3.50 indicates low decline; 3.51-4.00 indicates moderate decline; and 4.01-5.00 indicates a severe decline.

##### Physical Status

To measure the independence of ADL, the Katz Index of Independence in Activities of Daily Living is used [22]. The Katz Index of Independence in Activities of Daily Living is a questionnaire that aims to evaluate the following ADL: walking, feeding, dressing and grooming, toileting, bathing, and transferring. For each ADL, people with dementia are scored yes (1 point) or no (0 points) for independence in each of the 6 domains. A total score of 6 indicates full function, 4 indicates moderate impairment, and ≤2 implies severe functional impairment.

To assess instrumental ADL, the Lawton-Body instrumental ADL scale is used [23], which assesses the ability to manage finances, transportation, shopping and meal preparation, housecleaning and home maintenance, communication, and medications. People with dementia are scored ranging from 0 (low function, dependent) to 8 (high function, independent).

##### Experienced Burden by Informal Caregiver

To measure caregivers’ burden, the Dutch questionnaire “Ervaren Druk door Informele Zorg/Experienced Burden due to Informal Care” is used [24]. The Ervaren Druk door Informele Zorg/Experienced Burden due to Informal Care consists of 9 statements, whereby an informal caregiver rates these statements on a 5-point Likert scale ranging from No! to Yes! The answers “Yes!” “Yes,” and “Less or more” are scored with a 1, the answers “No!” and “No” are scored with a 0. The total score is calculated by adding the scores of the 9 statements and ranges from 0 to 9; this total score is interpreted as follows: 0-3, low burden; 4-6, moderate burden; and 7-9, high levels of burden.

##### Safety Issues

The informal caregiver is asked the following 3 (self-made) questions concerning the safety of people with dementia at home:

- Are there any people or agencies around the person with dementia who want to hurt him or her, profit from him or her, or make him or her anxious?
- Does the person with dementia sometimes think of harming him or herself or actually harming him or herself?
- Does the person with dementia ever cause unintended things that endanger him or her or his or her surroundings?

#### Interviews During Home Visits

After assessing complaints by questionnaires, which are mostly problem-oriented, an interview at home will be scheduled to qualitatively assess personal problems, needs, and wishes of people with dementia and informal caregivers. Textbox 1 provides an overview of the questions asked.

**Questions asked during the home-visit interview.**

- What is the impact of dementia on your life? What is the most urgent problem? What could help you to cope with the consequences of dementia?
- What things or tools did you think of yourself?
- For which problems, needs or wishes would you like to see a solution?
- Which activities do you like? What else can you enjoy?

#### Focus Groups Meetings

In the focus groups meetings, which are organized every second Tuesday morning of the month, a supervised group of, a minimal of 6 and maximum of 10 [25], people with dementia and their informal caregivers come together. In these meetings, participants explore the possibilities of developing innovations regarding their needs and wishes in coping in daily life and provide feedback to developers of the innovations. The qualitative information is merged into an anonymous summary and is communicated with participants and relevant stakeholders and stored in a database by GGzE.

#### Context-Field Studies

In the context-field studies, people with dementia and their informal caregivers have an opportunity to test a prototype of an innovation in the home setting for 2-3 weeks. AAfter testing, their experiences with regard to the prototype’s usability, feasibility and safety are evaluated. They receive a topic list with general and product-specific questions either digitally or by regular post (Textbox 2) and a home interview to evaluate that topic list is also scheduled. After multiple context-field studies of a prototype, the qualitative data are transcribed, and the written report is presented to participants and presented anonymously to the stakeholders of the prototype or design.

### Procedure

Every eligible person with the diagnosis of any form of dementia seen by case managers, specialized nurses, psychologists, or geriatricians receives oral and written information about ID 2.0 from an LL Leader (case manager or specialized nurse) at the GGzE. In addition, written information is shared by social media and at “Alzheimer Café” meetings. When interested, participants are contacted by phone by an LL Leader and additional information is provided about ID 2.0 and the LL design. Both people with dementia and informal caregivers are informed. Written informed consent is obtained from both by a case manager during a home visit when participants decide to collaborate as a dyad. The questionnaires (Table 3) are sent digitally or by post at the start of participation (T0), after 12 months (T1), and 24 months (T2).

In addition to completing the questionnaires, participants have an opportunity to take part in monthly “Focus group meetings,” which are co-creation sessions, in which people with dementia, informal caregivers, researchers, and stakeholders come together. The contents of the focus group meetings are driven by the needs of participants or by the input from stakeholders. These groups are open, which allows a continuous in- and outflow of participants.

**Questions asked during the evaluation interview of the context-field studies.**

- General questions
  - What was your expectation of the product?
  - In what kind of situations did you use the product?
  - Did using this product fulfill the need for example in agitation, safety and well-being?

- Product-specific questions
  - How often did you use this product on average per week?
  - How much time did you spend (in minutes) per week using this product?
  - Was your experience with this product pleasant?
  - Would you recommend this product?
  - Do you have suggestions for improvements?

**Table 3:** Questionnaires.

Outcomes	Questionnaire or instrument	At the baseline	1-year follow-up	2-year follow-up
Neuropsychiatric symptoms	Neuropsychiatric Inventory Questionnaire	✓	✓	✓
Subjective cognitive decline	Informant Questionnaire on Cognitive Decline in the Elderly	✓	N/A^a^	N/A
Level of independence in activities of daily living	Katz Index of Independence in Activities of Daily Living	✓	✓	✓
Level of independence in instrumental activities of daily living	Lawton-Body instrumental activities of daily living scale	✓	✓	✓
Caregiver burden	Ervaren Druk door Informele Zorg	✓	✓	✓
Safety	Three self-made items	✓	✓	✓

^a^N/A: not applicable.

Furthermore, participants also have an opportunity to evaluate designs of innovations in daily living in context-field studies. Participants reflect and evaluate the proposed design after testing it for 2-3 weeks in the home setting.

### Data Storage

The questionnaire data and the qualitative information obtained from home interviews, focus group meetings, and context-field studies are anonymously coded and uploaded by the team members of ID 2.0 and stored in a secured database stored at GGzE.

### Planned Statistical Analyses

For ID 2.0, new longitudinal quantitative and qualitative data will be collected and used to answer both scientific and clinically relevant questions. The longitudinal quantitative data will be analyzed using multilevel analysis, which enables the inclusion of all available data (ie, also those from participants with missing data). The predictive value of the determinants for the primary outcome measures at T1 and T2 will be determined using multivariate regression analysis (2 time-points). SPSS Statistics 24 will be used for these statistical analyses.

To analyze the qualitative data responses from the interviews, focus groups and context-field studies will be transcribed. After transcribing, the raw data will be uploaded to Atlas.ti, which enables a systematic content analysis by following the procedures of qualitative analysis according to Polit and Beck [26].

### Ethical Considerations

This protocol was approved by the Institutional Review Board of the GGzE. In addition, this protocol was approved by the Ethical Review Board of Tilburg University, Tilburg, the Netherlands. Written informed consent is obtained from all participants, in accordance with the Declaration of Helsinki (Seoul Revision, 2008) and the General Data Protection Regulation (AVG) [27].

Participants are given an opportunity to consult an independent professional about participating in the study and can withdraw at any time. The data are stored anonymously, and only team members of ID 2.0 have access. This initiative is conducted in accordance with the “Medical Research Involving Human Subjects Act” (Wet Medisch-Wetenschappelijk Onderzoek met mensen or WMO).

### Dissemination

The results obtained will be disseminated to the scientific and general public by publication in national and international (peer-reviewed) scientific and professional journals, as well as through presentations at conferences or meetings and the ggzei website [28]. The data will not be made public, assuring the study participants’ privacy. Request for data sharing will be considered on an individual basis.

## Results

The ID 2.0 initiative will be launched in September 2018 and is an ongoing research line. Our target population is people with dementia living in the community and their informal caregivers. The first results of the data analysis are expected in the spring of 2019.

## Discussion

By actively involving people with dementia and their informal caregivers during the process of developing interventions designed to meet their needs, newly designed concepts are more able to answer any unmet needs as dementia progresses [29-31]. ID 2.0 has high practical relevance for people with dementia and their informal caregivers; designers and innovators; formal caregivers; and researchers. For designers and innovators, the feedback of people with dementia and their informal caregivers is highly valuable in enhancing the usability of their innovations in daily living. By involving them in each phase of the developmental process, innovators are then able to make the required adjustments during this process based on the comments and suggestions of people with dementia and their informal caregivers. Eventually, these efforts will result in products and services that are more adaptable and usable for these people in their daily lives. An LL design can have commercial value for stakeholders by helping alleviate the risk involved when launching a new product or service [32]. Second, by gaining insight into people with dementia and their caregivers’ needs, designers and innovators become (more) aware in which areas of daily living future innovations are needed. Furthermore, the longitudinal design of ID and the multiple assessments not only provide a broad range of data about neuropsychiatric symptoms, (subjective) cognitive decline, and problems in the daily functioning of people with dementia but also about the burden informal caregivers experience. These data can be useful in both clinical practice and research. Professional caregivers gain more insights into the daily problems of people with dementia as dementia progresses and can, subsequently, adapt their care based on these insights. Future research could potentially use these valuable data to enhance health care for this vulnerable group in the community.

People with dementia and their caregivers might find this initiative time-consuming and could increase the burden they experience and lead to a potentially high percentage of dropouts during this longitudinal initiative, which could lead to challenges when attempting to compare data over time. However, attempts have been made to reduce the number of questions asked, and all measurements (except for the focus group meetings) take place in the home setting. In addition, participation in this initiative can be experienced as positive and fulfilling, as several studies have reported that people with dementia say that they want to be useful in society, to have a chance to voice their meaning (and be heard), and that they find it very rewarding if their experiences can benefit others [33-35]. People with dementia and their informal caregivers may not personally benefit from their own efforts during the development of the assistive technologies because products are not always immediately available for purchase. However, ID 2.0 actively informs people with dementia and their informal caregivers about new developments and products in the field in a very hands-on way. Another limitation of this longitudinal initiative is the degree of subjectivity of the obtained information, which might be viewed as negatively influencing the validity of the quantitative analyses. However, the ultimate goal of ID 2.0 is to provide valuable insights about the highly variable progression in personal needs and problems during the process of dementia; therefore, personal information is essential. By creating a sustainable collaboration with active user involvement, the 2 worlds of design and daily practice can finally come together and hopefully benefit the people who need help the most.

## Abbreviations

ADL: activities of daily living
ID 2.0: Innovate Dementia 2.0
GGzE: Geestelijke Gezondheidszorg Eindhoven en de Kempen (Mental Health Care Institute Eindhoven)
LL: Living Lab
NPI-Q: Neuropsychiatric Inventory Questionnaire
WMO: Medical Research Involving Human Subjects Act (Wet Medisch-Wetenschappelijk Onderzoek met mensen)
